# Benzodiazepines and Z-hypnotics consumption in long-COVID-19 patients: Gender differences and associated factors

**DOI:** 10.3389/fmed.2022.975930

**Published:** 2022-09-08

**Authors:** Pilar Carrasco-Garrido, Cesar Fernández-de-Las-Peñas, Valentín Hernández-Barrera, Domingo Palacios-Ceña, Isabel Jiménez-Trujillo, Carmen Gallardo-Pino

**Affiliations:** ^1^Department of Medical Specialties and Public Health, Faculty of Health Sciences, Universidad Rey Juan Carlos, Madrid, Spain; ^2^Department of Physical Therapy, Occupational Therapy, Rehabilitation and Physical Medicine, Faculty of Health Sciences, Universidad Rey Juan Carlos, Madrid, Spain

**Keywords:** long-COVID-19, benzodiazepine, Z-hypnotics, gender, predictors

## Abstract

**Background:**

Psychotropic drug consumption has increased during the COVID-19 pandemic. We describe here the prevalence and identifying factors associated with Benzodiazepine (BZD) and Z-hypnotics use among a sample of Spanish adults suffering from long-COVID-19 syndrome, from a gender perspective.

**Materials and methods:**

Data were anonymously collected between 15th December 2021 and 15th March 2022. The collection form consisted of several questions gathering sociodemographic information, post-COVID symptom, health profile, and pharmacological drug intake. Using logistic multivariate regression models, we estimated the independent effect of each of these variables on self-medicated consumption. Three models were generated (female, male, and both gender).

**Results:**

Prevalence of BZD and Z-hypnotics use was 44.9% (46.5% for women; 37.8% for men). Zolpidem was the most consumed drug among male (20.7%), and lorazepam in female (31.1%). Patterns of drug consumption among female were related with number of post-COVID symptoms and smoking habit (AOR 2.76, 95%CI 1.16–6.52). Males under 40 years of age are more likely to consume BZD and Z-hypnotics (AOR 5.52, 95%CI 1.08–28.27).

**Conclusion:**

The prevalence of consumption of BZD and Z-hypnotics in those subjects with long-COVID-19 in our study reaches values of 44.9%. Women with long-COVID-19 declare a higher prevalence of consumption than men. Predictors of BZD and Z-hypnotic in men were, age and number of medication use. Smoking habit and the number of post-COVID symptoms were predictive variables in women.

## Introduction

The novel coronavirus disease 2019 (COVID-19) has become a pandemic affecting health and wellbeing globally. Between its first appearance in December 2019 and the present date, 483 million of persons around the world have had a confirmed diagnosis of coronavirus 2019 disease (COVID-19) and more than six million have died (1).

As the pandemic continues, many patients who have overcome the acute phase of the disease are finding several difficulties to resume their day-to-day activities, since they present continuous symptoms, such as fatigue, dyspnea, muscle weakness and impaired quality of life. There is evidence suggesting that 75–80% of COVID-19 survivor’s exhibit post-COVID sequelae (2–4), which has been called long-COVID or post-COVID-19 Syndrome (5).

Since the pandemic began, the impact of the COVID-19 situation and its consequences on mental health has been investigated (6–8). Some authors have typified COVID-19 as a “psychiatric epidemic” (9). These studies show that anxiety symptoms, sleep disorders, psychological distress and depressive disorders have increased as a response to COVID-19 outbreak. Evidence indicates that there are gender differences in depression, anxiety and sleeplessness due to the pandemic with women experiencing higher depression and anxiety levels than men (10–12). Gender is an important determinant of mental health and the management of it in health services. Health surveys highlight the higher prevalence of poor mental health in women of all age groups and from all social groups. Diagnoses of depression and anxiety are more frequent among women, and the prescription of psychoactive drugs is significantly higher (13). All these factors indicate the existence of a process of medicalization of women’s mental health, although the interpretation of its origin is complex, since processes of overdiagnosis and overprescription can undoubtedly occur among them, but perhaps also of underdiagnosis and underprescription in men. The Spanish Mental Health Survey during the COVID-19 pandemic showed that, from the beginning of the pandemic, a total of 6.4% of the Spanish population consulted a mental health professional. A total of 44% of these individuals suffered from anxiety symptoms, 35% suffered from depression symptoms, and most of them were women (13).

Alongside this situation, and related to it, psychotropic drug consumption has increased during the COVID-19 pandemic, as reflected in different studies carried out in the United States and Europe. Such studies noted a rise in Benzodiazepine (BZD), antidepressant and Z-hypnotic prescriptions among the general population. Benzodiazepines are hypnotic drugs that enhance the activity of gamma-aminobutyric acid (GABA) at the GABAA receptor. This medication is mostly prescribed for anxiety disorders and as adjuvant therapy for depression associated with anxiety. On the other hand, Z-drugs are non-benzodiazepine hypnotics sharing a similar mode of action, but they are chemically distinct. The usual indication for Z-drugs is the first-line pharmacological strategy for the treatment of insomnia as alternative to benzodiazepines because of their more selective hypnotic profile and shorter elimination half-life values. These active drugs can generate tolerance and dependence, making it necessary to prolong treatment by using increasingly higher doses, despite the risks of long-term use. This circumstance is particularly relevant in view of the high consumption of medications in the elderly population, among whom hypnotics and anxiolytics are regularly used at a very high intake prevalence, often for long periods of time, and not always properly prescribed (14). These studies obtained different use rate values but had a common denominator: women show a higher use of these medications than men (15–17).

It is important to note that psychotropics are the type of drug showing greatest differences between women and men in terms of consumption and affectation in all age groups, therefore a gender perspective is needed in investigating these drugs’ use characteristics.

Despite this situation, there is currently little scientific evidence about consumption patterns of these psychotropic drugs among the long-COVID-19 syndrome population. These subjects show a high risk of presenting anxiety and depression symptoms (18), being female a risk factor for developing long-COVID-19, including depression, quality of sleep and mood disorders (19).

In this context, our study would be the first investigation aiming at describing the prevalence and identifying factors associated with BZD and Z-hypnotic use among a Spanish population suffering from long-COVID-19 syndrome, from a gender perspective.

## Materials and methods

### Study design

This pharmacoepidemiological study used a web-based cross-sectional survey design. A 39-questions survey asking for post-COVID symptoms and the use of pharmacological drugs was specifically developed by the research team and uploaded to the Google Forms platform (Google LLC, Mountain View, CA, United States). The survey was on-line and able to be completed by patients from 15th December 2021 to 15th March 2022.

### Participants

Participants were recruited from two Spanish long-COVID patient organizations (*Covid persistente España* and *Covid persistente Madrid including patients from Central, Central-South and Central-Nord regions of Spain)* by social networks and internet platforms for COVID-19 patient support. The survey inclusion criteria were COVID-19 survivors aged over 18 reporting post-COVID symptoms for at least three months after infection. SARS-CoV-2 infection should had been confirmed by previous positive reverse -transcription-polymerase chain reaction (RT-PCR) test from a nasopharyngeal and/or oropharyngeal swab, or from posteriori serological test that was positive for SARS-CoV-2 antibodies. Before starting the survey, participants electronically agreed an informed consent explaining the study and that its procedures were conducted in line with the Helsinki Declaration and the current Spanish Law on digital information (Law 14/2007 of Biomedic Investigation, Royal Decree 223/2004, General Data Protection Regulation (UE) 2016/679, and Standard Law 3/2018, December 5th, on Data Protection and Digital Rights). No monetary or non-monetary incentives were offered for voluntary completion of the survey.

### Survey

A pilot study with 20 individuals with long-COVID-19, not included in the final sample, was conducted to evaluate the comprehensiveness of the survey. This pilot study demonstrated that participants took 15 min to complete the survey. No potential item was excluded, since all pilot study participants properly understood and answered all questions.

The first part of the survey collected demographic data (age, gender, nationality, marital status, educational level, occupational status, and monthly income) and clinical data related to pre-existing medical comorbidities (with z medical diagnosis). The second part listed the following post-COVID symptom: dyspnea, fatigue, anosmia, ageusia, hair loss, chest pain, diarrhea, skin rashes, palpitations, brain fog, ocular disorders, cough, and concentration loss. Additionally, we also asked for the presence of psychological/emotional disorders such as depression, anxiety, and insomnia. Participants marked all symptoms that they perceived at the time of the survey and that had started after SARS-CoV-2 infection (categorized into <7; 7–10; >10). The following variables consisting of a number of chronic conditions (categorized into none; 1–2; ≥3), alcohol consumption (dichotomous variable Yes/No), and smoking (categorized into Smoker; Ex-smoker; Non-smoker) over the previous 30 days were also analyzed. In this section, participants likely were asked about their perceived health-related quality of life with the following possible answers: “Good/Poor or very poor.”

Finally, the third part of the survey focused on pharmacological intake. We considered as dichotomous dependent variables Yes/No replied to the following questions: *Have you taken tranquilizers, sedatives or sleeping pills in the last 30 days* and *were they prescribed for you by a doctor*? Participants answering “Yes” then marked the type of tranquilizer, sedative or sleeping pill that they consumed i.e., BZD or Z-hypnotics, by using their commercial names: Bromazepam, Alprazolam, Lorazepam, Diazepam, Potassium clorazepate, Zolpidem, and Lormetazepam.

To gain information about medications use, a question about the number of medications consumed other than BZD and Z-Hypnotics in the past 30 days (categorized into none; 1–2; ≥3) was formulated.

### Statistical analysis

Data was analyzed with Stata statistical software (Stata Corp., College Station, TX, United States, Stata/SE 16).

We calculated the prevalence rates for use of BZD and Z-hypnotics according to the study variables, with all data analyses performed separately for women and men. In addition, this gender-sensitive analysis had to question the gender relations that underlie and were reflected in the data (20). Pearson’s χ^2^-test was used for the bivariate comparison of proportions between gender with statistical significance set at *p* < 0.05 (two-tailed).

To estimate the independent effect of each study variable on the consumption of BZD and Z-hypnotics, we obtained the corresponding adjusted odds ratio (AOR) and 95% Confidence Interval (CI) via multivariate logistic regression analysis according to Hosmer et al. (21). Three models were generated, two by gender and the third as a total sample. All variables showing a significant association within the bivariate analysis were included in the multivariate analysis at the first step along with those variables considered relevant in the scientific literature, such as gender and age. At the second step, the model chose those independent variables into a parsimonious model with the least number of variables but with the higher significance (21).

## Results

The sample consisted of 391 respondents, mostly women (81%); 63.35% were between 40 and 50 years old (mean age: 47.6, SD: 8.75); the majority (53.3%) were married or in a stable relationship; employed people represented 51.8% of the sample; 51.5% reported having consumed alcohol and 9.0% were smokers. Data revealed that 44.9% of subjects reported having taken BZD and Z-hypnotics in the past 30 days.

It should be noted that 72.8% of study subjects had a negative perception of their state of health. As shown in Table 1, there were statistically significant differences by gender for all the variables.

**TABLE 1:** Sample characteristics by gender for the total survey sample.

	Male (*N* = 74)		Female (*N* = 316)		Total (*N* = 390)		*P*-value
				
	N	%	N	%	N	%	
**Age group**							0.603
<40 years	13	17.81	53	17.15	66	17.28	
40–55 years	43	58.90	199	64.40	242	63.35	
>55 years	17	23.29	57	18.45	74	19.37	
**Nationality**							
Immigrants	1	1.35	5	1.58	6	1.54	0.884
Spanish	73	98.65	311	98.42	384	98.46	
**Marital status**							
Single	29	39.19	92	29.11	121	31.03	0.037*
Married or couple	41	55.41	167	52.85	208	53.33	
Divorced/widow	3	4.05	51	16.14	54	13.85	
**Educational level**							
Primary school	6	8.11	12	3.80	18	4.62	0.159
Secondary school	24	32.43	87	27.53	111	28.46	
Higher education	44	59.46	217	68.67	261	66.92	
**Occupational status**							
Employed	42	56.76	160	50.63	202	51.79	0.003*
Unemployed	15	20.27	26	8.23	41	10.51	
Inactive	11	14.86	84	26.58	95	24.36	
**Monthly income**							
<1000 €	4	5.41	25	7.91	29	7.44	0.253
1000 €-2000 €	22	29.73	107	33.86	129	33.08	
>2000 €	43	58.11	146	46.20	189	48.46	
**Alcohol consumption in the past 12 months**							
No	30	40.54	159	50.32	189	48.46	0.131
Yes	44	59.46	157	49.68	201	51.54	
**Smoking habit in the past 12 months**							
Smoker	2	2.70	33	10.44	35	8.97	0.109
Ex-smoker	34	45.95	130	41.14	164	42.05	
Non-smoker	38	51.35	153	48.42	191	48.97	
**Number of chronic conditions**							
0	28	37.84	83	26.27	111	28.46	0.025*
1–2	26	35.14	95	30.06	121	31.03	
≥ 3	20	27.03	138	43.67	158	40.51	
**Number of post-COVID symptoms**							
<7	18	24.32	77	24.37	95	24.36	0.878
7–10	35	47.30	158	50.00	193	49.49	
>10	21	28.38	81	25.63	102	26.15	
**Self-assessment of health status**							
Good	17	22.97	89	28.16	106	27.18	0.662
Poor	35	47.30	138	43.67	173	44.36	
Very poor	22	29.73	89	28.16	111	28.46	
**Hospitalization in preceding 12 months**							
No	47	63.51	236	74.68	283	72.56	0.053
Yes	27	36.49	80	25.32	107	27.44	
**Admission to intensive care in preceding 12 months**							
No	64	86.49	312	98.73	376	96.41	<0.001*
Yes	10	13.51	4	1.27	14	3.59	
**Number of medications consumed other than BZD and Z-drugs in the past 30 day**							
None	5	6.76	19	6.01	24	6.15	0.900
1–2	32	43.24	130	41.14	162	41.54	
≥3	37	50.00	167	52.85	204	52.31	
**BZD and Z-hypnotics use in the past 30 days**							
No	46	62.16	169	53.48	215	55.13	0.177
Yes	28	37.84	147	46.52	175	44.87	

*p-value: Statistically association on comparing the distribution of variables between male and female. *p* < 0.05.

**FIGURE 1:**
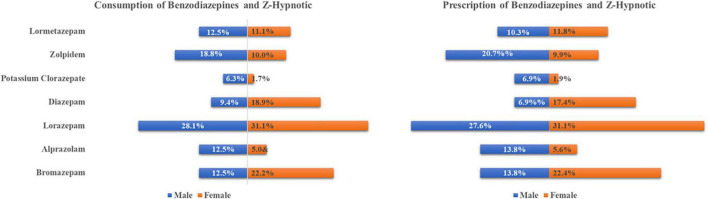
Prevalence of Benzodiazepine (BZD) and Z-Hypnotic use in patients with long COVID-19 by gender and generic drug.

The prevalence of BZD and Z-hypnotics consumption of long-COVID-19 subjects in Spain was 44.9% (46.5% women; 37.8% men). The prevalence of self-medication with these psychotropic drugs was 11.3%. Figure 1 visualizes prescribed and self-medicated consumption for differing active ingredients. Differentiated consumption is shown for these substances, with males making more use of Zolpidem (20.7%), and females showing a greater use of Lorazepam (31.1%).

Table 2 presents prevalence data for both genders according to sociodemographic variables, health profile and use of healthcare resources. Women who consumed alcohol and tobacco present higher levels of BZD and Z-hypnotics use than men (45.2% vs. 29.55% for alcohol; 60.5% vs. 50% for smoking). BZD and Z-Drug use among women who have more than 10 post-COVID symptom was 71.6% (*p* = 0.042). When subjects had a negative self-perception of their health, consumption values were higher (56.7%).

**TABLE 2:** Prevalence of BZD and Z-hypnotics consumption in patients with long COVID-19 according to sociodemographic, health profile and use of healthcare resources variables.

	Male (*N* = 28)		Female (*N* = 147)		Total (*N* = 175)		*P*-value
				
	N	%	N	%	N	%	
**Age group**							
<40 years	9	69.23	20	37.74	29	43.94	0.048*
40–55 years	13	30.23	99	49.75	112	46.28	0.022*
>55 years	6	35.29	26	45.61	32	43.24	0.453
**Nationality**							
Immigrants	1	100.00	5	100.00	6	100.00	NA
Spanish	27	36.99	142	45.66	169	44.01	0.180
**Marital status**							
Single	10	34.48	37	40.22	47	38.84	0.581
Married or couple	15	36.59	83	49.70	98	47.12	0.134
Divorced/widow	2	66.67	23	45.10	25	46.30	0.479
**Educational level**							
Primary school	1	16.67	7	58.33	8	44.44	0.117
Secondary school	10	41.67	51	58.62	61	54.95	0.143
Higher education	17	38.64	89	41.01	106	40.61	0.770
**Occupational status**							
Employed	12	28.57	71	44.38	83	41.09	0.067
Unemployed	7	46.67	13	50.00	20	48.78	0.837
Inactive	7	63.64	42	50.00	49	51.58	0.399
**Monthly income**							
<1000 €	2	50.00	17	68.00	19	65.52	0.488
1000 €-2000 €	10	45.45	48	44.86	58	44.96	0.959
>2000 €	15	34.88	66	45.21	81	42.86	0.231
**Alcohol consumption in the past 12 months**							
No	15	50.00	76	47.80	91	48.15	0.825
Yes	13	29.55	71	45.22	84	41.79	0.065
**Smoking habit in the past 12 months**							
Smoker	1	50.00	20	60.61	21	60.00	0.768
Ex-smoker	10	29.41	70	53.85	80	48.78	0.013*
Non-smoker	17	44.74	57	37.25	74	38.74	0.398
**Number of chronic conditions**							
None	11	39.29	36	43.37	47	42.34	0.705
1–2	9	34.62	35	36.84	44	36.36	0.834
≥3	8	40.00	76	55.07	84	53.16	0.211
**Number of post-COVID symptoms**							
<7	6	33.33	15	19.48	21	22.11	0.208
7–10	12	34.29	74	46.84	86	44.56	0.179
>10	10	47.62	58	71.60	68	66.67	0.042*
**Self-assessment of health status**							
Good	5	29.41	33	37.08	38	35.85	0.547
Poor	12	34.29	62	44.93	74	42.77	0.258
Very poor	11	50.00	52	58.43	63	56.76	0.476
**Hospitalization in preceding 12 months**							
No	16	34.04	106	44.92	122	43.11	0.172
Yes	12	44.44	41	51.25	53	49.53	0.541
**Admission to intensive care in preceding 12 months**							
No	23	35.94	147	47.12	170	45.21	0.104
Yes	5	50.00	0	0.00	5	35.71	NA
**Number of medications consumed other than BZD and Z-Hypnotics in the past 30 days**							
None	0	0.00	5	26.32	5	20.83	NA
1–2	10	31.25	52	40.00	62	38.27	0.363
≥3	18	48.65	90	53.89	108	52.94	0.564

*p-value: Statistically association on comparing the distribution of variables between male and female. *p* < 0.05.

Table 3 presents the findings for the three multivariate logistic regression analyses, which show the independent effect of each variable adjusted for the other variables on psychotropic medication use in our sample. The analysis indicates that there are no significant differences for BZD and Z-hypnotics use between males and females (AOR 1.32, 95%CI 0.75–2.31).

**TABLE 3:** Multivariable logistic regression analysis of factors associated with BZD and Z-Hypnotics consumption in patients with long COVID-19.

	Male	Female	Both gender
			
	AOR (95% CI)	AOR (95% CI)	AOR (95% CI)
**Age group**			
>55 years	1	1	1
40–55 years	1.06 (0.30–3.80)	1.36 (0.70–2.62)	1.22 (0.69–2.16)
<40 years	5.52 (1.08–28.27)	0.73 (0.31–1.72)	1.20 (0.57–2.54)
**Smoking habit in the past 12 months**			
Non-smoker	1	1	1
Smoker	ns	2.76 (1.16–6.52)	2.34 (1.05–5.24)
Ex-smoker	ns	1.80 (1.05–3.06)	1.38 (0.86–2.2)
**Number of post-COVID symptoms**			
	ns	1.36 (1.23–1.51)	1.26 (1.16–1.37)
**Number of medications consumed other than BZD and Z-drugs in the past 30 days**			
**Female**	ns		1.32 (0.75–2.31)

AOR, adjusted odds ratio; CI, confidence interval; ns, non-significant association.

The analysis of the pattern of BZD and Z-hypnotics use in males revealed that the variables that were independently and significantly associated with a greater likelihood of use were: age range <40 years old (AOR 5.52, 95%CI 1.08–28.27) and the number of medications consumed other than BZD and Z-hypnotics in the past 30 days (AOR 1.44, 95%CI 1.05–1.98).

The variables that were significantly associated with pattern of BZD and Z-hypnotics use in females were smoker (AOR 2.76, 95%CI 1.16–6.52), ex-smoker (AOR 1.80; 95%CI 1.05–3.06) and the number of post-COVID symptoms (AOR 1.36, 95%CI 1.23–1.51).

## Discussion

Psychotropics medication use during COVID-19 pandemic has been described in different investigations. Our investigation offers new and essential information, since it is one of the first studies describing BZD and Z-hypnotics consumption patterns specifically among individuals with long-COVID-19, incorporating a gender perspective.

We found that 44.9% of long-COVID-19 subjects in have used BZD and Z-hypnotics, with women showing higher percentages. Benzodiazepine and Z-hypnotics use in the general population has significantly increased in Spain during the COVID-19 pandemic, with values ranging from 6.3 to 7.9% for anxiolytics, and 4.8% for hypnotic-Sedatives (22, 23). Data from the Spanish Agency of Medicine and Health Products (*Agencia Española de Medicamentos y Productos Sanitarios*, AEMPS) in a report on the use of anxiolytic and hypnotic drugs in Spain showed consumption increasing from 90.60 defined daily dose/1,000 inhabitants per day (DHD) in 2019 to 93.04 DHD in 2020 (24). When we compare this level with other countries, a US-cohort showed a rise in prescribed consumption of BZD (5.3%) and Z-hypnotics (1.4%) drugs at the start of the COVID-19 pandemic (15). In Europe, the results by Fernandez et al., using an online questionnaire to assess changes in illegal and legal psychoactive substances consumption habits among the Portuguese population during the COVID-19 pandemic, indicate that use of tranquilizers and anxiolytics increased by 10.8%, and use of sleep-inducing drugs by 7.8% (16).

A common factor in these investigations is that women present the highest consumption rates. Data in the literature is unequivocal about the association between long-COVID-19 and women (19, 25, 26). The inclusion of a gender perspective in psychodrug use has generated new ways to understand consumption patterns among female and male users (27–29). Given the gender differences in COVID-19 and in epidemic behaviors, all indicators used should be gender-stratified, as data invisibility by gender may well be negatively affecting women more than men (30). Regarding long-COVID-19 subjects in our study, although BZD and Z-hypnotics consumption prevalence among women is 46.5%, no significant differences were observed compared with men (37.8%) in the multivariate analysis.

Lorazepam prescribed use in Spain accounts for 25.1% of DHD in 2021, according to AEMPS data. Our study results indicate that the most consumed BZD in long-COVID-19 subjects of both genders is Lorazepam (31.1% female vs. 27.6% male). This aligns with the study by Sánchez Diaz et al., which aimed to analyze the usage progression of anxiolytics available in pharmacies from 2015 to 2020, as well as the possible impact of COVID-19 on the use of these drugs (22). However, it should be noted that this study made no differentiation by gender, and so it is not possible to determine differences between women and men consumption. Zolpidem is the Z-hypnotic most consumed by long-COVID-19 men in our study (20.7%), with women showing lower values (9.9%).

Benzodiazepines and Z-hypnotics are the most commonly used drugs for managing anxiety, insomnia and depressive disorders. Their prescription is rising (15) due to an increased prevalence of anxiety and depressive disorders during the pandemic, which has been reflected in population-based surveys, such as the one by Czeisler et al. among United States adults (31), with a 33% rise in the prevalence of anxiety and depression, or the cohorts study by Xie et al. (32) where the hazard ratios (HR) for anxiety and depressive disorders among COVID-19 subjects (compared with controls) were 1.35 (95%CI 1.30–1.39) and 1.39 (95%CI 1.34–1.43), respectively.

By identifying BZD and Z-hypnotics consumption patterns among the long-COVID-19 population, we find that only age acts as a predictor of usage for men. Therefore, subjects under 40 years are five times more likely to consume these drugs than older men). This effect was not observed in women with long-COVID-19. Different researches performed during the pandemic have demonstrated that psychotropic drug consumption rises with age, particularly after 65, in both men and women (15, 16, 23). One possible explanation for our results is that long-COVID-19 subjects in our study was a young population, with only 19% of the sample older than 55. A recent study performed by Kim et al. (33) to identify factors associated with long-term consequences derived from COVID-19 infection, carried out via an online questionnaire in a population of subjects in the same age ranges as the current study, also found no association of psychological symptoms, such as anxiety, with age in patients aged 50 or older (20.9% of the sample). They argued that other factors, such as social support and antecedents had a greater impact than biological age over time (33).

When we analyze the variables related to lifestyle, smoking (smoker and ex-smokers) presents the highest values of association to BZD and Z-hypnotic consumption in females with long-COVID-19. In a similar way to our results, the study by Schecke et al. examined whether legal and illegal psychoactive substance use changed among German women during the COVID-19 pandemic. That survey also showed that the logistic regression for generalized anxiety acted as a significant predictor for increased use of nicotine and other substances (12). This increase could be a reaction to the stress derived from the pandemic situation, as has previously been demonstrated in the outcomes of other studies researches carried out in Europe (34, 35) and the United States (36). Smokers normally use certain drugs such as anxiolytics; there is a well-known close relationship between smoking and psychiatric disorders (a higher proportion of subjects with mental health disorders smoke, when compared to general population). Women smokers are a specific population with more severe mental health outcomes that need to be specifically addressed (37).

Although gender-related differences have been demonstrated in the acute phase of COVID-19, few studies have assessed such differences in long-COVID-19 (19, 28, 38, 39). These investigations shown that women demonstrated a wider symptomatology than men and that long-COVID-19 predictors are different between men and women (38). Likewise, women in our study who demonstrated a greater number of post-COVID symptoms have a greater probability of consuming BZD and Z-hypnotics. Gender is an important determinant in long-COVID-19 because it is a significant predictor for persistent symptoms in women. However, to date, there is no clear explanation of why women show a greater probability of experiencing COVID-19 long-lasting symptoms. Different explanations have been proposed, ranging from various physiological factors, such as a greater prevalence of pain syndromes in women, factors related to higher depressive levels, poor sleep quality, etc. along with factors such as isolation, stress and inactivity (19, 39). In this context, BZD and Z-hypnotic use in females with long-COVID-19 who demonstrated several symptoms would be justified by their greater rate of affective disorders, or their greater vulnerability in the society. This could possibly be due to a greater predisposition among women to recognize and express their symptomatology, thus making all the health inequalities produced by the generation of gender social construction become evident.

Multivariate analyses indicate that men with long-COVID-19 who declared drug use in the last month show a greater probability of using BZD and Z-hypnotics. We cannot rule out the possibility that male participants had a more serious medical history of the disease. This may be related to the fact that men generally only seek healthcare services, included medication consumption, when a health problem is perceived and considered limiting (27).

Although one strength of our study is that it is one of the first to identify factors associated with BZD and Z-hypnotics consumption among subjects with long-COVID-19, it also has some limitations. First, respondents with more severe long-COVID-19 may have a higher tendency to actively participate in the survey. Additionally, current data are based on a sample of 391 Spanish individuals with long-COVID-19. In fact, the sample of men included in the survey was small and some comparisons could have been underpowered stratifying by gender. Future population-based studies including large samples are needed to confirm or refute current results. Second, the inherent limitation of surveys on medication consumption lies in the cross-sectional nature of the study data, which prevents from determining the direction of the associations identified. Longitudinal studies collecting data at different follow-up periods will permit to identify prolonged consumption of these medications. Third, as the current study was an online survey study, information about persistent symptoms or medication use was self-reported directly by respondents and was not based on objective evaluation. Fourth, the fact that the data was self-reported means the prevalence values obtained to draw up the profiles of BZD and Z-hypnotic use may be underestimated: potential sociocultural characteristics surrounding psychotropic use may have led to some individuals with long-COVID-19 being reticent about openly admitting to BZD and Z-hypnotic consumption. We cannot exclude that patients were taking any other sleeping agents/herbal medicines or psychotropics with sedative effects since our survey focused on BZD and Z-hypnotic use. Finally, it is important to consider that our data are based on a sample of patients where was unable to determine the SARS-CoV-2 variant of infection, if participants have been reinfected or which type of vaccine (if any) have received. Additionally, it should be considered that our sample only included individuals with a previous confirmation of SARS-CoV-2 acute infection by RT-PCR or serological test.

## Conclusion

The prevalence of consumption of BZD and Z-hypnotics in subjects with long-COVID-19 in our study reaches values of 44.9%. Women with long-COVID-19 show a higher prevalence of consumption of these medications than men, although these differences were not statistically significant. Differentiated gender consumption of these drugs were observed. Lorazepam is the BZD most used among women, while Zolpidem is the Z-hypnotic most frequently used by men.

Among men in our study, variables such as age <40 years and the number of medications consumed in the past 30 days were associated with a greater likelihood of BZD and Z-hypnotic consumption. Smoking and the number of post-COVID symptoms act as predicting variables for BZD and Z-hypnotic consumption in women with long-COVID-19.

It is, therefore, important to know how COVID-19-related mitigation measures are associated to psychotropic drugs prescription patterns, commonly used to treat conditions related to mental health, and to get a better understanding of the link between gender and prescribed psychotropic drug types. The inclusion of gender perspective contributes to improving that knowledge, as it is a key step in reducing the adverse effects of untreated or undertreated mental health conditions.

## Data availability statement

The raw data supporting the conclusions of this article will be made available by the authors, without undue reservation.

## Ethics statement

The studies involving human participants were reviewed and approved by Local Ethic Committees of Universidad Rey Juan Carlos (URJC0907202015920). The patients/participants provided their written informed consent to participate in this study.

## Author contributions

PC-G and CF-d-l-P originated and designed the study and coordinated the writing of the manuscript. VH-B contributed to the analysis of the data and drafting of the manuscript. PC-G, CF-d-l-P, VH-B, DP-C, IJ-T, and CG-P contributed to the interpretation of the results and drafting of the manuscript. All authors had full access to all the data in the study and took responsibility for the integrity of the data and the accuracy of the data analysis, and approved the final version.
